# A Novel Body Weight–Supported Postural Perturbation Module for Gait and Balance Rehabilitation After Stroke: Preliminary Evaluation Study

**DOI:** 10.2196/31504

**Published:** 2022-03-01

**Authors:** Amanda Meyer, Henry Charles Hrdlicka, Erica Cutler, Jill Hellstrand, Emily Meise, Kaitlyn Rudolf, Pete Grevelding, Matthew Nankin

**Affiliations:** 1 Department of Inpatient Occupational Therapy Gaylord Specialty Healthcare Wallingford, CT United States; 2 Milne Institute for Healthcare Innovation Gaylord Specialty Healthcare Wallingford, CT United States; 3 Department of Inpatient Physical Therapy Gaylord Specialty Healthcare Wallingford, CT United States; 4 Frank H Netter MD School of Medicine Quinnipiac University North Haven, CT United States

**Keywords:** stroke rehabilitation, postural balance, gait and ambulation, balance perturbation, postural perturbation, body weight support system, occupational therapy, physical therapy, long-term acute care hospital, Berg Balance Scale, Activities-Specific Balance Confidence Scale

## Abstract

**Background:**

Impaired balance regulation after stroke puts patients and therapists at risk of injury during rehabilitation. Body weight support systems (BWSSs) minimize this risk and allow patients to safely practice balance activities during therapy. Treadmill-based balance perturbation systems with BWSSs are known to improve balance in patients with age- or disease-related impairments. However, these stationary systems are unable to accommodate complex exercises that require more freedom of movement.

**Objective:**

This study aims to evaluate the effect of a new balance perturbation module, which is directly integrated into a track-mounted BWSS, on balance impairments secondary to acute stroke.

**Methods:**

This unblinded quasi-randomized controlled preliminary study was conducted in a rehabilitation-focused long-term acute care hospital. Participants were recruited from stroke rehabilitation inpatients with an admission Berg Balance Scale (BBS) score of 21 (out of 56) or greater. Over a 2-week period, consented participants completed 8 BWSS or BWSS with perturbation (BWSS-P) treatment sessions; study activities were incorporated into regular treatment to avoid disruption of their normal care. Although both groups conducted the same balance and gait activities during their treatment sessions, the BWSS-P sessions included lateral, anterior, and posterior balance perturbations. Pre- and postintervention BBS and Activities-Specific Balance Confidence (ABC) assessments were the primary outcome measures collected. Institutional BBS data from the year before installation of the track-mounted BWSS were retrospectively included as a post hoc historical standard of care comparison.

**Results:**

The improved postintervention BBS and ABC assessment scores showed that all participants benefited from therapy (*P*<.001 for all pre- and postintervention comparisons). The average BBS percent change for the BWSS-P sample (n=14) was 66.95% (SD 43.78%) and that for the BWSS control sample (n=15) was 53.29% (SD 24.13%). These values were greater than those for the standard of care group (n=30; mean 28.31%, SD 17.25%; *P*=.02 and *P*=.005 respectively), with no difference among the BWSS groups (*P*=.67). ABC score changes were also similar among the preintervention and postintervention BWSS groups (*P*=.94 and *P*=.92, respectively).

**Conclusions:**

Both BWSS groups demonstrated similar BBS and ABC score improvements, indicating that balance perturbations were not detrimental to postacute stroke rehabilitation and were safe to use. These data provide strong rationale and baseline data for conducting a larger follow-up study to further assess if this new perturbation system provides additional benefit to the rehabilitation of gait and balance impairments following stroke.

**Trial Registration:**

ClinicalTrials.gov NCT04919161; https://clinicaltrials.gov/ct2/show/NCT04919161

## Introduction

### Background

Each year, more than 795,000 people experience a stroke [1]. Stroke, or cerebral vascular accident, is a neurological event that can lead to devastating physical and cognitive deficits, such as the inability to ambulate, impaired balance regulation, loss of coordination, and impaired communication [2]. Because of the physical and cognitive deficits experienced following a stroke, many patients require admission to an inpatient rehabilitation facility with the goal of maximizing their independence before returning to the home setting [3]. Gait dysfunction is a common secondary impairment of stroke that usually requires specific rehabilitative actions [4].

Following a stroke, patients are often observed via motion analysis to navigate obstacles more conservatively and with abnormal gait patterns [5]. This is likely associated with the loss of muscle strength secondary to stroke, which could increase the risk of falling [5]. Within 6 months of discharge, falls occur in up to 70% of patients following a stroke, highlighting the importance of focusing on improving patients’ balance and gait during the early rehabilitation phase [6].

It is estimated that over 90% of stroke survivors would report that the fear of falling would negatively impact their performance in daily living activities [7]. Fear of falling has been shown to influence balance and gait control in older adults, supporting the theory that balance and gait should be considered during rehabilitative methods [7]. These psychological factors are also strong predictors of falling compared with physical factors or the presence of pathology. Patient self-assessments can be important indicators of fall risk, as patients may better understand their capabilities and limitations than what the physical tests demonstrate [8].

The ability to walk, stand, climb stairs, and other mobility-related functional tasks are critical components in achieving functional independence. However, following a stroke, it is often difficult for patients with balance impairments to safely practice balance and gait training without putting both therapists and patients at risk for injury. Incorporating robotic technologies for neurological rehabilitation can play a critical role in delivering safe and effective gait and balance therapy [9].

The integration of body weight support systems (BWSSs) following a stroke, spinal cord injury, or other neurological disorders has continued to expand over the last 2 decades [10]. The range of tools available to therapists to treat patients with these impairments continues to grow [10]. Using BWSSs to unload paretic lower limbs, patients with gait impairments can practice a higher repetition of steps in a safe and controlled manner. As the patient performs gait training, these systems support the patient’s body weight. This permits those with excessive weakness and poor coordination to minimize the risk of injurious falls and to ambulate and perform more intensive therapy sessions sooner in their recovery.

In addition to BWSSs, balance perturbation systems improve gait and balance control after stroke, or in response to other age- and disease-related balance impairments. This is accomplished by purposefully unbalancing patients so that they can rehabilitate their postural control [11-16].

### Objectives

In this study, we evaluate the efficacy of a recently developed, not yet reported, balance perturbation module for the ZeroG BWSS. This new balance perturbation training module is directly integrated into the BWSS and allows therapists to induce safe lateral, anterior, and posterior perturbations via a Wi-Fi–enabled handheld device. During both stationary and ambulatory activities, this system was used to unbalance participants to train their reactive balance control and balance reactions, including ankle, hip, and stepping strategies [17]. The purpose of this preliminary study was three-fold: (1) evaluate the safety and feasibility of using this technology in the clinical setting, (2) develop a practical protocol for clinical use, and (3) determine whether there is any evidence to suggest that this newly developed BWSS balance perturbation system provides additional benefits to patient gait and balance rehabilitation after stroke over the standard BWSS protocol without perturbations, which would support further investigation of the technology.

## Methods

### Research Design

This was an unblinded quasi-randomized parallel active comparator–controlled preliminary study conducted at Gaylord Specialty Health Care (Wallingford, Connecticut, United States), a long-term acute care hospital (LTACH). As a result of an oversight in the requirements for clinical trial registration and the definition of an applicable clinical trial [18], the authors humbly admit there was a delay in clinical trial registration for this study. However, we are pleased to report that the educational and procedural issues leading to this oversight have been rectified and that the study has been retrospectively registered as follows: ClinicalTrials.gov NCT04919161 [19].

### Ethical Considerations

Before participant recruitment, the study was reviewed and approved by the hospital’s Institutional Review Board to ensure the study complied with the ethical standards set by the Declaration of Helsinki and CONSORT (Consolidated Standards of Reporting Trials) 2010 (Multimedia Appendix 1) [20]. All patients provided informed consent.

### Participants

All participants were admitted to the LTACH under the inpatient stroke rehabilitation program after receiving a stroke diagnosis at a regional acute care hospital. Participant recruitment occurred over 12 months (October 2019 to September 2020). Patients admitted to the inpatient stroke rehabilitation program were evaluated by physical and occupational therapy within the first 72 hours of admission, at which point an initial Berg Balance Scale (BBS) score was obtained as appropriate. To be considered, patients had to score ≥21 on the BBS during their initial physical therapy evaluation. As defined by Berg [21], with a BBS score of ≥21, patients were considered to have a *fair global balance* rating and could walk with assistance. In this context, a fair balance rating was interpreted to be equivalent to a moderate fall risk [21]. Patients who did not meet these inclusion criteria during their initial evaluation were able to screen-in later, pending BBS reassessment. If the reassessment showed sufficient functional improvement (ie, BBS ≥21) and the patient’s planned discharge date was at least two weeks after the reassessment, the patient was approached for study recruitment.

In addition to meeting the BBS score criteria, participants needed to be ≥18 years of age, be able to understand and respond to simple verbal instructions in any language, and be able to tolerate and actively participate in at least three, 30-minute, weekly sessions in the BWSS. Patients were ineligible to participate if they did not meet any one of these criteria or presented with 1 or more of the exclusion criteria shown in Textbox 1.

**Exclusion criteria for study participation.**

**Exclusion criteria**

- Cognitive deficits that would disrupt the ability to provide informed consent
- Berg Balance Scale score <21
- Active seizure
- Spinal stabilization requiring use of Halo device
- Uncontrolled hypertension
- Uncontrolled hypotension
- Unstable skin structures (ie, skin grafts and chest tubes)
- Unstable rib or lower extremity fractures
- Osteoporosis
- Active enteric infection control precautions
- New limb amputations
- Need for >50% high flow oxygen
- Bodyweight of more than 450 pounds (204 kg), that is, the structural limitation of the body weight support system

**Figure 1:**
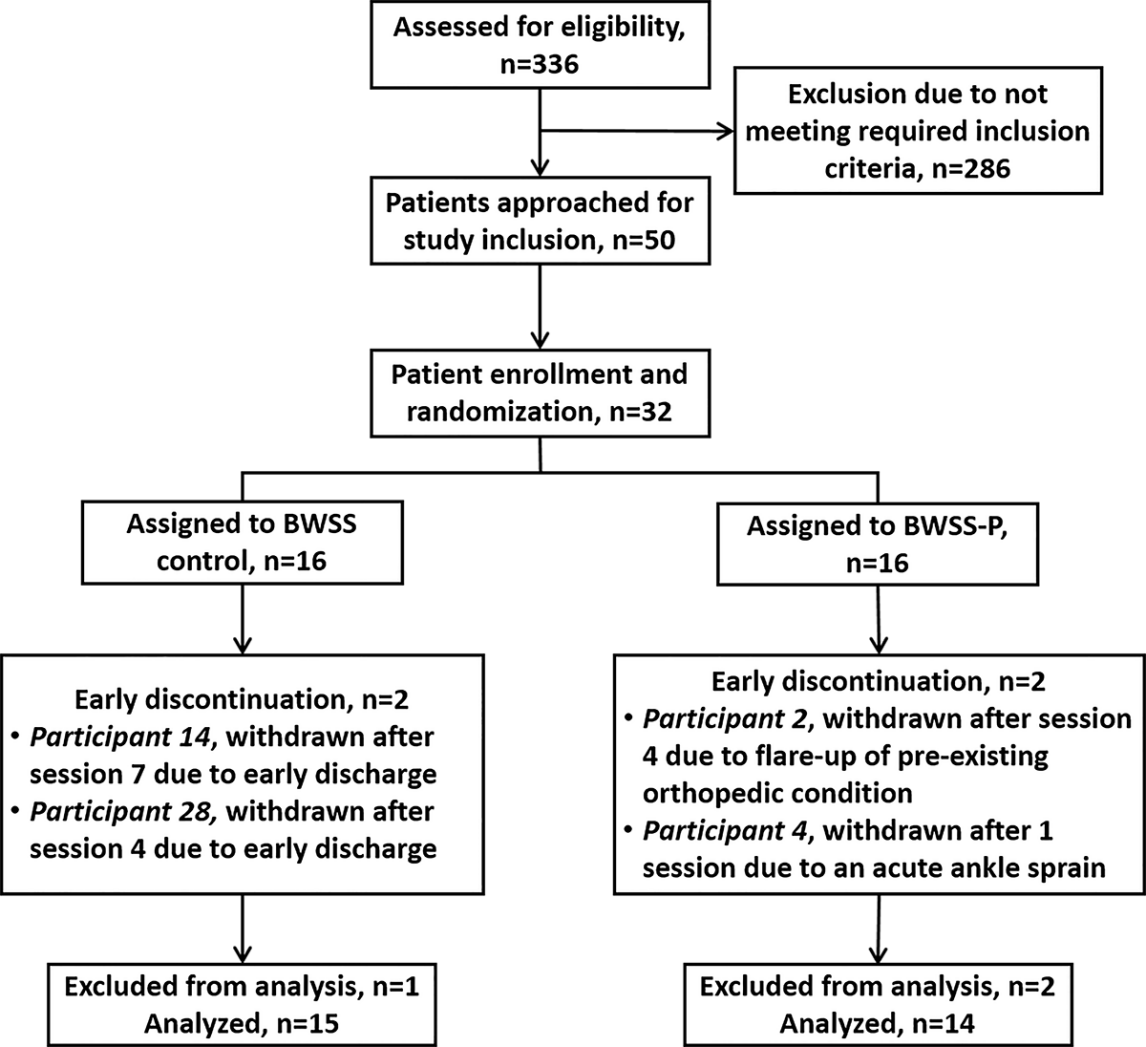
Participant flowchart. Of the 336 patients admitted for stroke rehabilitation that were assessed for study eligibility, 14.9% (50/336) were approached for study inclusion. Ultimately, 64% (32/50) of participants were enrolled in the study and assigned to either the body weight support system (BWSS) control or body weight support system with perturbation (BWSS-P) groups. During the study, 13% (4/32) of participants withdrew from the study early; 50% (2/4) because of early discharge, 25% (1/4) because of a flare-up of a pre-existing orthopedic condition, and 25% (1/4) because of an acute ankle sprain. Data from 9% (3/32) of participants was excluded from the final analysis.

After providing informed consent, participants were assigned in an alternating fashion by the investigators to either the BWSS control or BWSS with perturbation (BWSS-P) group. To our knowledge, this is the first instance of this technology being studied. As such, we targeted a convenience sample of at least 30 participants for preliminary evaluation. Of the 50 patients approached for inclusion, 32 (64%) were enrolled, and 29 (58%) completed the study (Figure 1).

Patients admitted for acute stroke rehabilitation typically received 2-5 hours of skilled rehabilitative services 5-6 days per week, including physical, occupational, and speech therapies and therapeutic recreation. All participants enrolled in this study were deemed appropriate to receive this level of care.

### Outcome Instruments

The BBS and the Activities-Specific Balance Confidence (ABC) scale were the primary study end points. Both assessments have been validated for use in the inpatient stroke population and have high interrater reliability [22-25]. The BBS is a standardized balance assessment that uses various balance tasks to objectively measure a person’s balance and determine if a participant is at low, moderate, or high fall risk. The ABC scale is a 16-item patient-reported outcome measure that subjectively measures one’s self-perceived balance confidence. The ABC scale achieves this by asking the user to consider various hypothetical situations and tasks and if they could perform them without losing balance or experiencing a sense of unsteadiness; it is based on a rating scale from 0% (no confidence) to 100% (completely confident) [8,23].

To identify eligible candidates for the study, chart reviews were regularly conducted to collect the admission BBS scores of patients with stroke, who were recently admitted. The progression of patients who were disqualified from the study because of their admission BBS scores were tracked through periodic chart reviews to determine if they had sufficiently improved to be approached for study recruitment.

During regular treatment, a 10-point modified functional independence measure (mFIM) was used to assess each participant’s assistance needs when ambulating and undergoing toilet transfers. On the basis of the original 7-point functional independence measure (FIM) [26], the mFIM was developed by our LTACH institution to better describe the progress of our patient population. The mFIM is similar to the traditional FIM in that it ranges from dependent to independent and is used to score patients in various functional domains, including ambulation and transfers. The mFIM differs by subdividing the original FIM category of *minimal assistance (category 4)* into *minimal assistance (category 4)* and *contact guard assistance (category 5).* The original FIM category of *supervision (category 5)* is also subdivided into *close supervision (category 6)*, *supervision (category 7)*, and *distant supervision (category 8)*. The criteria used to grade each of the 10 points can be found in Table S1 in Multimedia Appendix 1.

A final chart review was conducted at the end of the study to collect the participants’ BBS and mFIM scores from their physical therapy discharge documentation. The ABC scale was administered by site investigators at the time of consent and immediately after the last intervention session.

Additionally, BBS scores of patients admitted to the same stroke rehabilitation program between October 2017 and August 2018 were retrospectively collected for post hoc analysis as a historical standard of care (SOC) comparison. These retrospectively collected scores were filtered to remove initial BBS scores <21 and those collected after the launch of the BWSS at our institution in September 2018. This resulted in the inclusion of retrospective BBS data from 30 patients who were not treated with BWSS. Similar historical data were not available for ABC assessment or mFIM scores.

### BWSS Equipment

For this study, the BWSS used was the Food and Drug Administration listed ZeroG Gait and Balance System (Aretech, LLC) [27]. Unlike some BWSSs, this device is mounted on an overhead track that follows patients as they ambulate, maintaining a vertical direction of unloading via the tether [27,28]. Like other BWSSs, this system is designed to unload patients of up to 200 pounds (91 kg) of body weight, while simultaneously protecting them from falling. Unlike some other systems, this device maintains the preset amount of body weight support even if there is a change in vertical displacement by the patient, that is, when navigating stairs or sitting down. For this study, only 10 pounds (4.5 kg) of participants’ body weight—the minimum weight required to engage the BWSS—was continuously unloaded for participants in both the control and intervention groups. The rationale for using only 10 pounds (4.5 kg) was to minimize unloading effects and balance support provided by the straps during balance perturbation. However, if a participant were to fall, the system would still detect the change, decelerate the fall, and stop the descent after a set distance. The fall distance was set between 8 and 12 inches for this study.

Unlike other BWSSs, a newly developed balance perturbation module, known as the training response in postural rehabilitation (TRiP), is directly integrated into the ZeroG BWSS. This perturbation module is different from other systems as the balance perturbations are elicited directly through the BWSS and do not require a treadmill [11-14], tilt-table, shaking platform [14,15], or manual exertion by a therapist [16]. Further, they can be induced during normal gait and balance exercises during therapy.

### BWSS and BWSS-P Exercises and Interventions

The BWSS control group interventions consisted of various balance activities, including marching, side-stepping, retro-ambulation, step-taps, and step-ups. The BWSS control group also practiced various gait tasks, including ambulation over the ground, going up and down stairs, and performing sit-to-stand transitions. The BWSS-P intervention group performed the same balance and gait activities as the control group with the addition of left and right lateral, anterior, and posterior perturbations.

Assistive devices and equipment were used during intervention sessions as recommended by the participant’s primary therapist to facilitate ambulation, including canes, rolling walkers, hemi-walkers, ankle-foot-orthoses, ankle support braces, and upper extremity slings. The goal of using assistive devices was to only facilitate ambulation and *real-life* function and not necessarily protect against balance perturbations in the BWSS-P group. We believe that delivering balance perturbations while using assistive devices are transferable and appropriate challenges of real-life functions and are an important component of rehabilitation and recovery. Of note, although assistive devices were used during the study sessions, they were not used during the BBS assessments.

In the absence of balance perturbations, the BWSS motor remained positioned vertically above the participants as they moved along the track, ambulating, or performing other exercises with the investigator. As participants moved in line with the track, therapists used a Wi-Fi–enabled handheld device linked to the BWSS to elicit anterior or posterior balance perturbations during ambulation. These were induced by causing the BWSS motor to either rapidly accelerate ahead of or decelerate and reverse behind the participant. The resulting force of this acceleration or deceleration caused balance perturbation. Left and right lateral perturbations were similarly induced while the participant was in a static stance positioned under and perpendicular to the track. As demonstrated in video examples provided by the manufacturer, the rapid movement of the overhead component allowed little time for participants to prepare for the oncoming perturbation [29]. Participants in the BWSS-P group experienced 8 total perturbations in each session, 2 in each of the 4 directions described above.

All BWSS-P participants started at perturbation level *one* and progressed up to a maximum perturbation level of *ten* through the course of the study. The amount of force exerted at each perturbation level was preset by the manufacturer. The perturbation level (ie, intensity or force) used in each session was based on the participants’ progress and observational analysis made by the therapist from the participants’ responses to the perturbation level. If a participant was able to tolerate the initial perturbation level without exhibiting an appropriate balance reaction (including absent or aberrant ankle, hip, or stepping strategies), the perturbation level was incrementally increased until an appropriate balance reaction was exhibited [17]. If a participant was unable to recover and elicited a fall response in the system, the perturbation level was decreased by 1 level to ensure patient safety and the exercise was repeated to reinforce the exercise mechanics and participant confidence. The highest perturbation level was recorded after each session.

Participants in both study groups received 8 treatment sessions over 2 weeks. As necessary, participants received up to 2 sessions in 1 day to ensure they completed the required 8 sessions before discharge. To be pragmatic and not disrupt participant care, study sessions were incorporated into the participants’ regular care. At our institution, treatment sessions were broken into 30-minute blocks. This time included patient transportation, equipment setup, and for this study, donning the BWSS harness. In general, participants received 20 minutes of active time in the BWSS for each 30-minute treatment block. All sessions were analyzed equally, despite possible variations in the length of time the participants were in the BWSS.

### Data Analysis

Data were analyzed using GraphPad Prism (version 9.0.0; GraphPad Software). To compare the observed proportion of male and female in the BWSS groups, Fisher exact test was used. Additionally, the odds ratios (ORs) for the proportions and the respective 95% CIs (Baptiste Pike testing) were calculated. Participant age was also compared between groups using the nonparametric Mann–Whitney *U* test was used.

When data from multiple time points and 2 or more groups were present, we used a 2-way mixed effects model analysis of variance (ANOVA). This was to evaluate for the presence or absence of time effects independent of treatment modality, treatment modality effects independent of time, and the effect of time and treatment modality combined. Šídák multiple comparison test was then used to calculate all the in-group and between-group comparisons. The BBS, ABC, toileting transfer mFIM, and ambulation mFIM before and after intervention scores were included in this analysis.

To account for baseline BBS score differences among the BWSS, BWSS-P, and SOC groups, the degree of change for each participant was also calculated using *percent change* as follows: ([Postintervention – Preintervention] / Preintervention) × 100.

Whereas calculating the straight score change would lose information about the preintervention scores, this normalization strategy minimizes the amount of information lost by returning the degree or amount of change made by each individual relative to their preintervention score. Although BBS percent change was normally distributed for each group (Shapiro–Wilk test; SOC, *P*=.12; BWSS, *P*=.39; BWSS-P, *P*=.37), the SDs significantly differed (Brown–Forsythe test for variance; *P*<.001). As such, a 1-way Brown–Forsythe ANOVA and Dunnett T-3 multiple comparisons test for BBS percent change group comparisons was used.

To evaluate changes in the perturbation level progression, an ordinary 2-way ANOVA with Šídák multiple comparisons test was used for the entire data set. Subsets (low, moderate, and high responders) were analyzed using paired Kruskal–Wallis ANOVA with Dunn multiple comparison test.

For data represented as a box plot, each box represents the median and the 25% and 75% quartiles, respectively. The whiskers extend 1.5 and −1.5 of the IQR, respectively, triangle symbols reflect data points beyond the 1.5 IQRs, and the *+* symbol represents the arithmetic mean.

## Results

### Participant Characteristics

Of the 29 participants who completed the treatment course, 15 (52%) were alternately assigned to the BWSS control group and 14 (48%) were alternately assigned to the BWSS-P group. In the BWSS group, 87% (13/15) were men and 13% (2/15) were women **(**Table 1**)**. In the BWSS-P group, 71% (10/14) were men and 29% (4/14) were women **(**Table 1**)**. A participant in the BWSS control group did not complete the eighth and final session because of an early discharge; however, the data from their 7 completed sessions were included in the analysis. Compared with the control group, the BWSS-P group was similarly aged (*P*=.92; Table 1). Using Fisher exact test, we also observed similar proportions of men and women (*P*=.39). This was also reflected in the OR testing of the proportions (OR 2.6, 95% CI 0.47 to 15.30).

**Table 1:** Participant characteristics.

		BWSS^a^ control (n=15)		BWSS-P^b^ (n=14)		Group difference (95% CI)^c^	*P* value
		Participant, n (%)	Age (years), mean (SD; range)	Participant, n (%)	Age (years), mean (SD; range)		
**Cohort (** **N=29)**		15 (52)	57.8 (12.98; 46 to 78)	14 (48)	57.5 (14.24; 28 to 78)	−1.0 (−12 to 12)	.92
	Male	13 (87)	57.5 (12.53; 42 to 73)	10 (71)	57.4 (11.31; 41 to 78)	−1.0 (−13 to 11)	.76
	Female	2 (13)	60.5 (20.51; 46 to 75)	4 (29)	57.8 (22.25; 28 to 78)	−0.5 (−47 to 32)	.99

^a^BWSS: body weight support system.

^b^BWSS-P: body weight support system with perturbation.

^c^Nonparametric Mann–Whitney *U* test was used; group differences and reported 95% CI are based in differences of the medians.

Throughout the study, most participants tolerated the BWSS induced perturbations well. However, 2 (6%) of the 32 original participants enrolled in the BWSS-P group did not complete all 8 therapy sessions because of injury. A participant experienced an unexpected flare-up of a pre-existing chronic orthopedic condition unrelated to the BWSS perturbation module after session 4. A second participant had an acute ankle sprain during ambulation in the BWSS during session 1. The nature of this injury was deemed likely because of a combination of the BWSS perturbation module and ankle instability secondary to the participant’s stroke. A third participant also withdrew early from the study because of an early discharge after session 4. The data from these 3 individuals was excluded from analysis (Figure 1).

### BWSS Perturbation Level Progression

**Figure 2:**
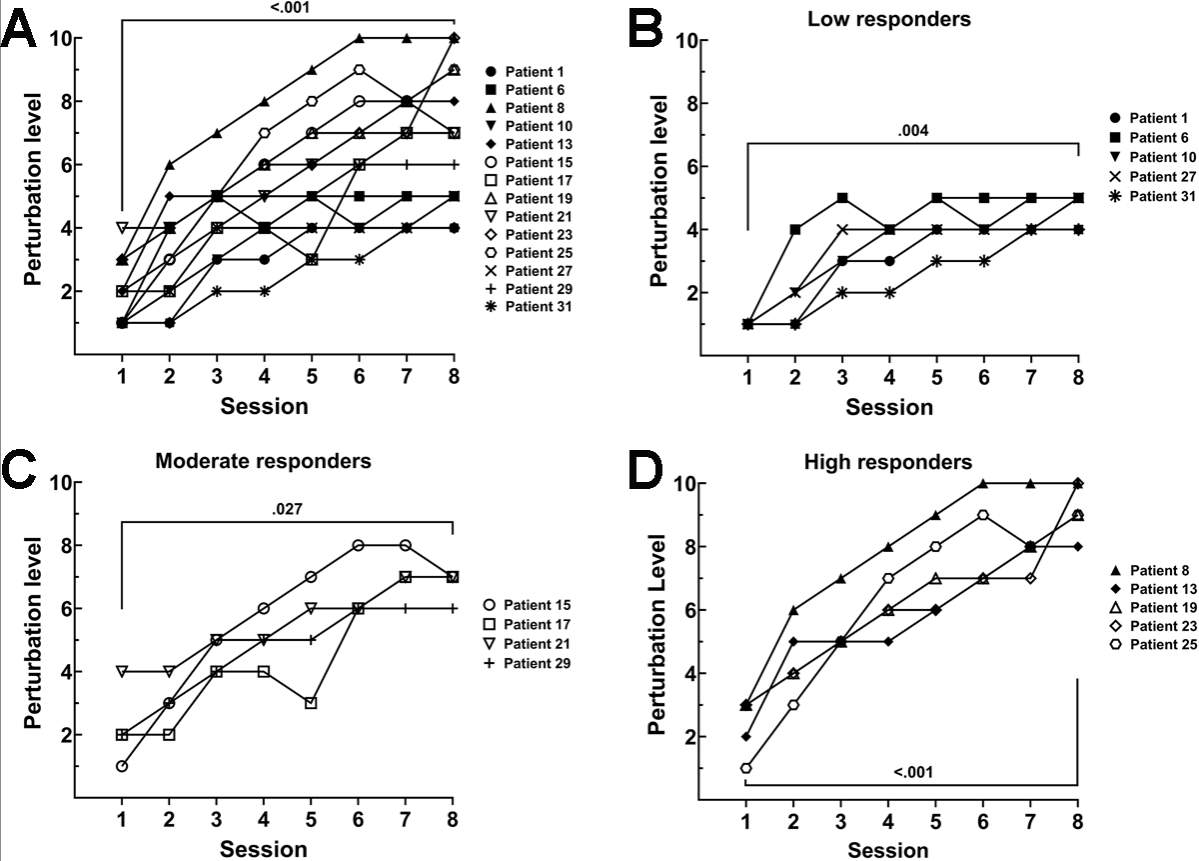
Perturbation level progression. From the body weight support system, the highest perturbation level achieved was recorded for each participant, after each therapy session. Each participant who completed the study successfully increased their perturbation level between the first and last study-related therapy session (A). The perturbation level progression for the participants that completed the study could be broken down into three categories: low responders (B), moderate responders (C), and high responders (D). *P* values shown are for the comparison of session 1 and session 8 perturbation levels.

From the BWSS perturbation module, the highest perturbation level achieved for each patient in each session was recorded. Although the final perturbation level achieved by the final session varied, all participants showed increases in perturbation level by the end of the study (*P*<.001; Figure 2A).

Interestingly, no statistical difference in perturbation level was observed between sessions 6, 7, and 8 (Table S2 in Multimedia Appendix 1). Further, these data can be divided into 3 categories. First, the *low responders* showed early perturbation level progression but plateaued early, peaking at perturbation levels 4-5 (Figure 2B). The *moderate responders* showed steady progress throughout the study, peaking between perturbation levels 6-8 (Figure 2C). The *high responders* rapidly progressed through the BWSS-P levels, peaking between BWSS-P levels 9-10 (Figure 2D).

### Evaluation of Participant BBS Scores

To evaluate pre- and postintervention BBS data (Table 2), a 3 column × 2 row 2-way mixed effects ANOVA was used. This analysis showed that there was a significant main effect (*F*_DFn, DFd_) associated with time (*F*_1,56_=283.5; *P*<.001) on BBS scores with grouped postintervention scores (mean 48.02) being greater than grouped preintervention scores (mean 33.61). Further, there was a significant main effect of treatment modality (*F*_2,56_=9.609; *P*<.001) on BBS scores, with the pooled group mean of the SOC (mean 45.35) being greater than the BWSS-P (mean 39.36) and BWSS (mean 37.73). Finally, there was also a significant interaction effect between time and treatment modality (*F*_2,56_=7.902; *P*<.001).

**Table 2:** Summary of Berg Balance Scale assessments.

Group	Preintervention, mean (SD; range)	Postintervention, mean (SD; range)	Score change^a^, mean (SD)	Percent change^b^, mean (SD)
SOC^c^ (n=30)	40.20 (7.66; 25-52)	50.50 (5.41; 33-56)	10.30 (5.11)	28.31 (17.25)
BWSS^d^ (n=15)	30.20 (6.41; 21-41)	45.27 (6.67; 34-54)	15.07 (5.61)	53.29 (24.13)
BWSS-P^e^ (n=14)	30.43 (7.97; 21-47)	48.29 (6.94; 35-56)	17.86 (8.57)	66.95 (43.78)

^a^Score change was calculated as (postintervention – preintervention).

^b^Percent change was calculated as (([postintervention – preintervention] / [preintervention]) ×100%).

^c^SOC: standard of care.

^d^BWSS: body weight support system.

^e^BWSS-P: body weight support system with perturbation.

**Figure 3:**
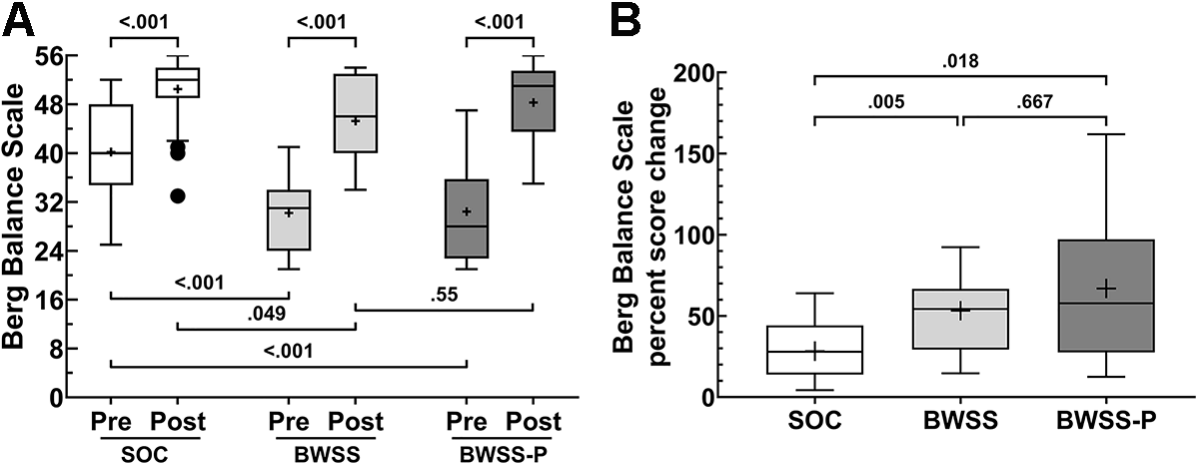
Berg Balance Scale assessment (BBS). Participant’s pre- and postintervention BBS assessment scores were used to track their improvement and response to the therapy. In addition to the body weight support system (BWSS) control and body weight support system with perturbation (BWSS-P) protocols, data from 2018, before the implementation of the BWSS, served as a historical standard of care (SOC) comparison group. Raw scores were first examined in aggregate (A). BBS percent change was calculated for each participant to show the magnitude of change between pre- and postintervention scores (B). In panel A, *P* values are shown only for comparisons that are significantly different or of clinical interest. Box plots represent the median and the 25% and 75% quartiles, respectively. The whiskers extend 1.5 and −1.5 of the IQR, respectively; circle symbols reflect data points beyond the 1.5 interquartile ranges; + symbols represent the mean; SOC: n=30, BWSS control: n=14 to 15, BWSS-P: n=13 to 14.

Šídák multiple comparisons test was used to determine if any in-treatment group comparisons were different. All in-treatment group comparisons were significantly different (*P*<.001), highlighting the time effect noted in the 2-way mixed effects ANOVA (Figure 3A; Table S3 in Multimedia Appendix 1). Evaluating the between-group comparisons at the 2 different time points, we observed that the mean preintervention BBS score of the SOC group was significantly higher than the mean preintervention BBS score of both the BWSS and BWSS-P groups (*P*<.001); the BWSS and BWSS-P preintervention BBS scores did not differ (*P*=.99). In addition, the mean postintervention BBS score of the SOC group was significantly higher than the BWSS mean postintervention BBS score of the BWSS group (*P*=.049) but not that of the BWSS-P group (*P*=.68); BWSS and BWSS-P postintervention BBS scores were not different (*P*=.55; Figure 3A; Table S4 in Multimedia Appendix 1).

We assessed the degree of change for each individual by calculating the percent change (Figure 3B; Table 2) and analyzing group differences using a 1-way Brown–Forsythe ANOVA (F*_DFn, DFd_). This analysis indicated that there were significant between-group interactions (F*_2.00, 23.4_=7.859; *P*=.003). Although the mean pre- and postintervention BBS scores were similar among groups, multiple comparison testing showed the percent change for the BWSS-P group (n=14; mean 67%, SD 43.8%) was greater than the SOC group (n=30; mean 28.3%, SD 17.3%; *P*=.02). The percent change in the BWSS control group (n=15; mean 53.3%, SD 24.1%) was also greater than that in the SOC group (*P*=.005). Although the percent change of the BWSS-P group was marginally greater than that of the BWSS control group, it was not significantly different (*P*=.67).

### Assessing Participant Functional Independence With Ambulation and Transfers

Using the institution’s mFIM scoring (Table S1 in Multimedia Appendix 1), participants’ functional independence during ambulation and toilet transfers at admission and discharge was assessed using a 2 column × 2 row 2-way mixed effects ANOVA.

For ambulation mFIM scores, where the mean ambulation assistance score increased in both the BWSS control (4.36, SD 1.03, to 7.80, SD 1.20) and BWSS-P treatment (4.75, SD 0.83, to 8.64, SD 0.93) groups, this analysis showed a significant time effect (*F*_DFn, DFd_) associated with mFIM ambulation scores (*F*_1,27_=257.9; *P*<.001), with grouped postintervention scores (mean 8.22) being greater than grouped preintervention scores (mean 4.54). Further, a significant treatment modality effect (*F*_1,27_=4.26; *P*=.049) associated with mFIM ambulation scores was observed, with the BWSS-P group mean (mean 6.70) being modestly greater than that for the BWSS group (mean 6.07). Finally, no significant interaction effects were observed between time and treatment modality (*F*_1,27_=0.99; *P*=.33) for ambulation mFIM scores.

Using Šídák multiple comparisons test, we observed that in-group preintervention versus postintervention comparisons were significantly different (*P*<.001). Both between-group preintervention ambulation mFIM scores (*P*=.47) and postintervention scores (*P*=.06) did not differ (Figure S1A in Multimedia Appendix 1; Tables S5 and S6 in Multimedia Appendix 1).

Similarly, the mean toilet transfer mFIM scores increased in both the BWSS control (4.30, SD 0.59 to 7.70, SD 1.16) and BWSS-P treatment (4.89, SD 0.79, to 8.39, SD 1.04) groups. The 2-way mixed effects ANOVA analysis showed a significant time effect (*F*_DFn, DFd_) on mFIM toilet transfer scores (*F*_1,27_=257.9; *P*<.001), with grouped postintervention scores (mean 8.05) being greater than grouped preintervention scores (mean 4.60). Further, there was a significant main effect of treatment modality (*F*_1,27_=5.79; *P*=.02) on mFIM toilet transfer scores, with BWSS-P group mean (mean 6.64) again being marginally greater than the BWSS group (mean 6.00). Finally, there was no significant interaction effect observed between time and treatment modality (*F*_1,27_= 0.05; *P*=.82).

Using Šídák multiple comparisons test, we observed that the in-treatment group preintervention versus postintervention comparisons were significantly different (*P*<.001). Both between-group preintervention scores (*P*=.17) and postintervention mFIM toilet transfer scores (*P*=.09) were not different (Tables S7 and S8 and Figure S1B in Multimedia Appendix 1).

### Measuring Participant Self-reported Balance Confidence

Participants’ self-confidence in performing daily tasks was also evaluated using the ABC scale. The mean ABC scores (%) increased in both the BWSS control (61.81, SD 22.55, to 82.38, SD 13.43) and BWSS-P treatment (63.88, SD 20.34, to 84.81, SD 11.52) groups. A 2-way mixed effects ANOVA identified a significant time effect (*F*_DFn, DFd_) on ABC scores (*F*_1,26_=34.26; *P*<.001), with grouped postintervention scores (mean 83.59) being greater than grouped preintervention scores (mean 62.85). Unlike the BBS and mFIM scores described up to this point, a significant treatment modality effect (*F*_1,26_=0.16; *P*=.69) was not observed, with the BWSS-P group mean (mean 74.35) being only marginally greater than the BWSS group (mean 72.09). Further, an interaction effect between time and treatment modality (*F*_1,26_=0.05; *P*=.82) was not observed (Table S6 in Multimedia Appendix 1) for the ABC scale score.

**Figure 4:**
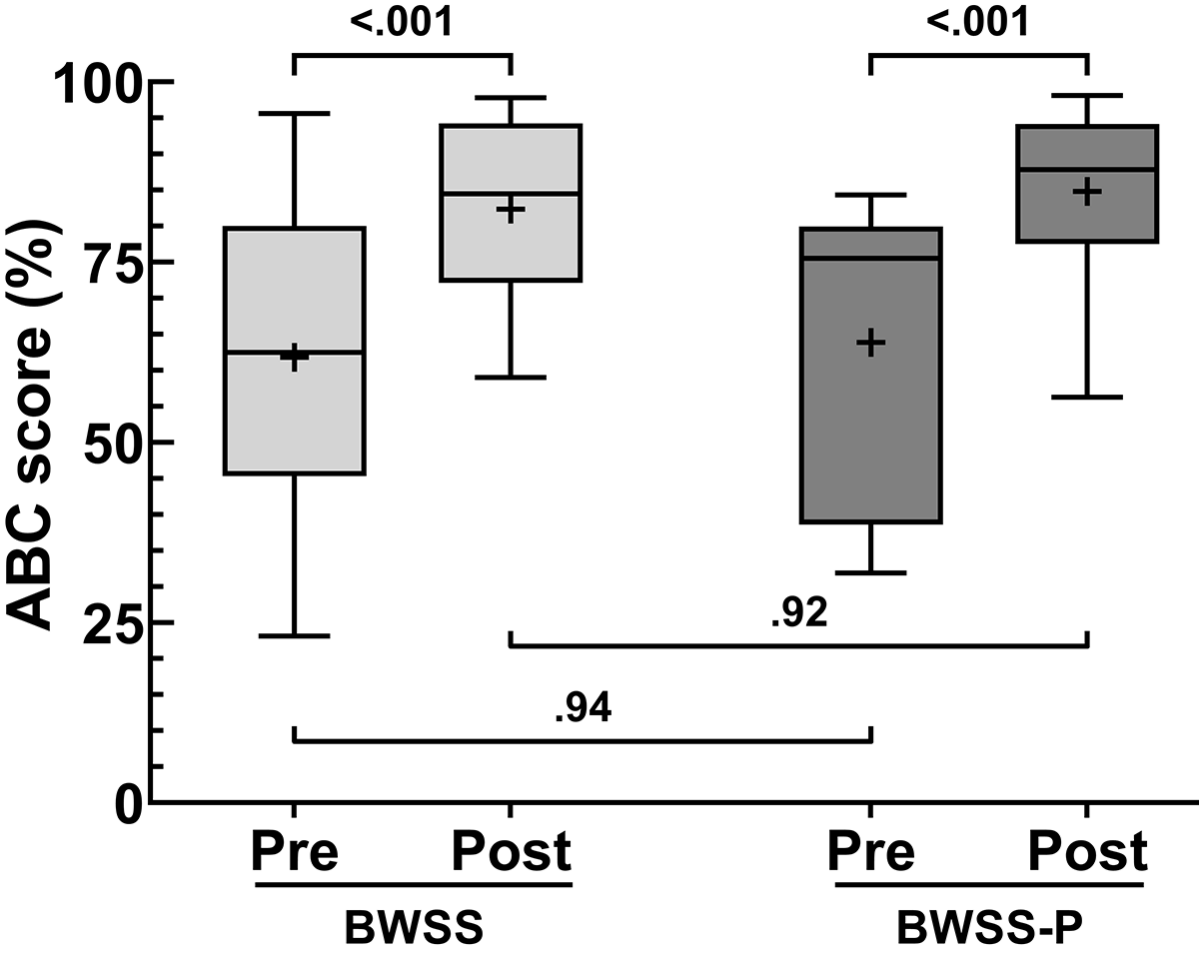
Activities-Specific Balance Confidence (ABC) scale assessment. The ABC scale was given to participants before and after the intervention to gauge their confidence in performing daily tasks. The box plot represents the median and the 25% and 75% quartiles, respectively. The whiskers extend 1.5 and −1.5 of the IQR, respectively; + symbols represent the mean; body weight support system (BWSS) control: n=14 to 15, body weight support system with perturbation (BWSS-P): n=13 to 14.

Using Šídák multiple comparisons test, we observed that in-group preintervention versus postintervention comparisons were significantly different for ABC scores (BWSS: *P*<.001; BWSS-P: *P*<.001). Between-group preintervention scores (*P*=.94) and postintervention scores (*P*=.92) were not significantly different (Figure 4; Tables S9 and S10 in Multimedia Appendix 1).

## Discussion

### Principal Findings

We conducted this preliminary study to evaluate the effectiveness of a new BWSS-integrated balance perturbation training module. If effective, this tool may be able to further improve patient balance after an acute stroke. This module induced controlled reactive and potentially anticipatory balance perturbations during normal gait and balance exercises without using a treadmill or other equipment. Participants in the BWSS and BWSS-P groups demonstrated similar improvements in BBS, ABC assessment, ambulation mFIM scores, and toileting transfer mFIM scores. This indicates that the BWSS-P protocol is not detrimental and may benefit postacute stroke rehabilitation. With retrospectively collected BBS data from 2018 serving as a retrospective post hoc SOC comparison group, both BWSS groups displayed greater BBS percent score changes than the SOC group. These data support the overall conclusion that this new BWSS balance perturbation module may help improve patient balance after acute stroke when following a prescribed treatment and rehabilitation plan. However, additional research is required to definitively determine the full benefits of this technology in rehabilitation.

Conventional balance perturbation training, including modified treadmills [11-14], tilt-tables [14,15], or external force provided by the therapist directly [16], may pose an injury risk to the therapist and the patient. In addition, if a patient experiences an injurious fall during treatment, it may further contribute to a fear of falling after stroke. Although the incorporation of BWSSs over treadmills decreases the injury risk, this is not representative of functional ambulation in patients’ homes or community environments [30,31]. Further, these strategies are stationary and limit the types of activities and exercises that can be performed during balance perturbation (eg, navigating a turn). Systems such as these may also limit the participation of some patients who would otherwise benefit from reactive balance perturbation training, such as those uncomfortable or unable to ambulate on a treadmill.

Therapists also have the option of inducing balance perturbations by manually exerting an external force (ie, pulling or pushing the patient) while a patient is in a BWSS. Although more accessible than using specialized equipment, both the application of force by the therapist and the amount of perturbation experienced by the patient are subjective and could be difficult to control and replicate consistently. Integration of the balance perturbation module in the BWSS described here resolves many of these issues, including allowing for freedom of movement to perform most gait and balance exercises in a dynamic environment, increasing the accessibility for eligible patients, and performing perturbations in a consistent, repeatable, and quantitative manner, while optimizing therapist and patient safety.

The ABC scale was used to determine how the BWSS-P would affect participants’ self-perceived balance confidence when asked to consider hypothetical scenarios that would challenge their balance. Interestingly, our analysis found that although a significant time effect was observed, an effect associated with treatment modality was not present. This suggests that BWSS and BWSS-P are equally effective in improving participants’ balance confidence. However, we were unable to determine whether these scores were better than those in the SOC group.

### Limitations, Caveats, and Considerations for Future Studies

As this was the first study to evaluate this novel technology, we can identify several limitations and caveats that need to be considered when interpreting the data and planning future studies.

In this study, we retrospectively included BBS data from the year leading up to the implementation of the BWSS. The rationale for this was to include these data as a post hoc historical SOC comparison group, representative of therapy with no BWSS. Although including a prospective *No BWSS* group would have led to cleaner comparisons, our stance is this would have been unethical, as it would have meant withholding care known to benefit patient outcomes. It is well documented that rehabilitative gait and balance training in BWSSs largely benefit patient rehabilitation. Therefore, we were unable to collect data for all outcomes of the SOC group, as they were not regularly collected during normal care (ie, ABC score) or were not readily available (ie, mFIM scores).

Further, the admission BBS scores of the SOC group were approximately 10 points higher than those of the BWSS groups because the preintervention scores started at a higher baseline; it was not unexpected that the postintervention BBS scores were higher. To account for this, we could have curated the SOC data set to be case-matched to the BWSS control group, so the range and mean of the preintervention scores were similar. However, to avoid any selection bias that might have been introduced, we normalized the data by calculating the percent change.

An important caveat to note is that the study activities were only a small part of the participants’ overall treatment strategy. Several factors, including physical therapy outside the study and natural progression, were likely to have contributed to improvements in patient status and function. As we were unable to control for what physical therapy (ie, gait and balance training) activities occurred outside of the study sessions, we included the BWSS control and historical SOC comparison groups.

Improvements to the BWSS perturbation module alone were made difficult, as both BWSS groups showed similar BBS score improvements. Although the mean scores were not significantly different, the variability of the initial BBS scores of the BWSS study groups may have limited our ability to accurately determine the impact of the perturbation module. This variability, in part, is reflective of the diverse patient population that was recruited; any qualifying inpatients with stroke and with a BBS of ≥21 were approached. Although calculating the percent change for each participant works to address this, this variability can be improved in one of several ways.

First, the data analysis could be stratified to compare the amount of change or improvement by admission BBS scores. This would allow us to better refine what populations benefit the most from this treatment. Second, an upper BBS score could be incorporated into the inclusion criteria. For example, for patients with an acute stroke, a BBS score of 45 (out of 56) has been used to describe normal functional ability after stroke [24]. Finally, a matched-control method could be implemented to ensure that the same range of initial BBS scores were represented in the BWSS groups. However, as described above, this strategy is not ideal, as it can introduce implicit selection biases. In any case, a larger population will be required in future studies to achieve the appropriate power needed to fully determine the impact of the BWSS perturbation module.

Variability in the timing of the postintervention BBS assessments may have also contributed to the lack of significant differences among the BWSS groups. The postintervention BBS scores were obtained by the participants’ primary physical therapist at the time of their discharge. Most participants had discharge dates close to the last session of the study intervention. However, this does not account for any progress the participant might have made after the last session leading up to their discharge date, especially if there were unexpected delays to discharge. To address this in future studies, we propose delivering a separate BBS assessment within 48 hours of the last session, if the participant’s discharge assessment was not already collected during that time.

Most participants completed the study-related sessions over a 2-week period; however, this study was partly conducted during the early months of the COVID-19 pandemic (March-August 2020). This environment may have shortened the time that eligible patients were willing to spend in the inpatient setting if they were able to safely navigate the home environment with the assistance of family members. As a result, many patients who met the inclusion criteria for the study did not remain inpatients long enough to receive the required 8 sessions. As a further consequence of expedited discharge dates because of COVID-19, 34% (10/29) of the participants, at least once, needed to receive 2 sessions per day to complete all 8 sessions; in 1 case, the patient was discharged before they were able to complete the last treatment session. It is unclear if the increased intensity positively or negatively contributed to the rate of progress.

To address the possibility of irregular lengths of stay in the future, we propose evaluating and comparing the dose-response relationship of the balance perturbation module over 2-6 sessions, as well as the total time in the system. We feel it is rational to reduce the number of total sessions as there was no significant difference in perturbation level progression following session 6 (Table S2 in Multimedia Appendix 1) and it would open up the recruitment pool to eligible patients with a shorter length of stay and allow us to refine the optimal dosing. Additional studies could also investigate how many sessions per day and per week are most effective at improving balance control, reaction, and confidence.

Despite these limitations, this preliminary study was strengthened by the quasi-randomized controlled design and low participant dropout rate (3/32, 9%). Further, the incorporation of the post hoc historical SOC BBS data strengthened the study as it allowed for comparisons to be made with a population without BWSS treatment. With 52 years of combined physical therapy rehabilitation experience, the study was further reinforced by the advanced specialty and board certifications of the treating investigators.

This preliminary study allowed for the development of a feasible protocol and provided the preliminary data needed to calculate effect size, conduct power analysis, and estimate an appropriate sample size for future studies. With an appropriately powered sample size, we believe the effect of this BWSS-P protocol and technology on patient balance rehabilitation after stroke, compared with BWSS alone, could be better generalized than what we were able to conclude in this preliminary study. Furthermore, such studies could examine how other variables (ie, stroke location and other compounding diagnoses) impact patient progress and response to balance perturbation training. Incorporating additional dynamic gait assessments that more closely resemble functional movement patterns and reactive balance–specific outcome measures, such as the dynamic gait index or functional gait assessment [32,33], may also help us to better understand the full implications of this new balance perturbation module.

### Conclusions

This study has multiple implications for clinical practice in inpatient rehabilitation settings. The BWSS-P protocol positively impacted the balance performance of a subset of inpatients with stroke, who scored ≥21 on their BBS assessment. Not only did the BWSS-P improved participants’ balance and decreased their fall risk compared with the SOC and BWSS alone it also improved participants’ overall confidence and reduced their fear of falling, similar to that observed using the BWSS alone. As the prevalence of BWSS-integrated balance perturbation modules, such as the track-mounted ZeroG TRiP system, continues to grow, there will be a number of opportunities for continued research and development in this area.

## Appendix

Multimedia Appendix 1 Referenced supplemental materials and the completed CONSORT (Consolidated Standards of Reporting Trials) 2010 checklist.

## Abbreviations

ABC: Activities-Specific Balance Confidence
ANOVA: analysis of variation
BBS: Berg Balance Scale
BWSS: body weight support system
BWSS-P: body weight support system with perturbation
CONSORT: Consolidated Standards of Reporting Trials
FIM: Functional Independence Measure
LTACH: long-term acute care hospital
mFIM: modified functional independence measure
OR: odds ratio
SOC: standard of care
TRiP: training response in postural rehabilitation
