# Supplementary material for: A Novel Body Weight–Supported Postural Perturbation Module for Gait and Balance Rehabilitation After Stroke: Preliminary Evaluation Study
Source: JMIR Rehabil Assist Technol. 2022 Mar 1;9(1):e31504. doi: 10.2196/31504 (PMC8924779; doi:10.2196/31504)
Supplement: Multimedia Appendix 1 [file rehab_v9i1e31504_app1.pdf]

Supplemental Materials: Preliminary Evaluation of a Novel Body-Weight Supported Postural Perturbation Module for Gait and Balance Rehabilitation after Stroke

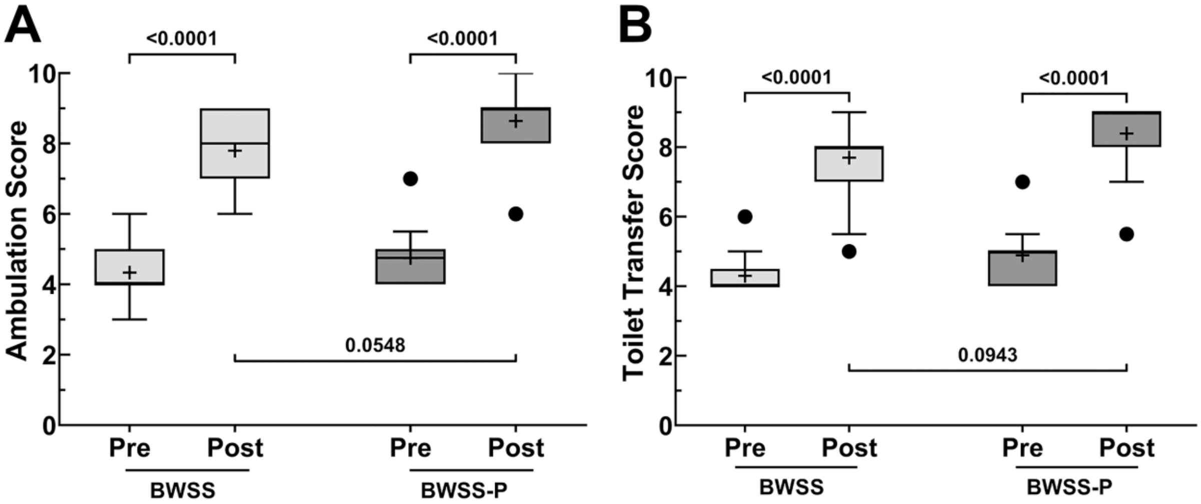

Supplemental Figure 1. Ambulation and Toileting Transfer Assistance mFIM Scores. In addition to evaluating patient functional outcomes by the BBS, we also measured the level of assistance participants required during ambulation **(A)** and toilet-transfer **(B)**. These were measured using the modified FIM scale shown in **Supplemental Table 1**. Only comparisons that are significant ($p<0.05$) or trending towards significance ($p<0.1$) are shown; all other comparisons are not significantly different. For a full summary of the pairwise comparisons, please refer to **Supplemental Tables 5-8**. The box-plot represent the median and the 25% and 75% quartiles respectively. The whiskers extend -1.5 and 1.5 of the interquartile range respectively; circle symbols reflect data-point values outside the interquartile range; **+** represents the mean; BWSS n=14-15, BWSS-P n=13-14

Supplemental Table 1. Modified functional independence measure (mFIM) definitions and criteria

Score	Descriptor [a, b]	Definition
1	Dependent (D)	Dependent mobility; subject/patient providing less than 25% of the work
2	Maximal Assistance (MAX)	Subject/patient performs 25 to 49% of the work
3	Moderate Assistance (MOD)	Subject/patient performs 50 to 74% of the work
4 [c]	Minimal Assistance (MIN)	Subject/patient performs 75 to 100% of the work
5 [c]	Contact Guard Assist (CG)	Subject/patient requires light hands on assistance for balance but no physical lifting is required
6 [d]	Close Supervision (CS)	Subject/patient requires the therapist to be close by in case the patient experiences a loss of balance, but does not provide physical or hands on assistance
7 [d]	Supervision (S)	During supervision the therapist is proving supervision at more than an arm length away.
8 [d]	Distant Supervision (DS)	This is "intermittent supervision." The therapist does not have to be in the room.
9	Modified Independence (MOD I)	The subject/patient is independent WITH use of adaptive device, techniques, or increased time.
10	Independent (I)	The subject/patient is independent WITHOUT use of adaptive device, techniques, or increased time.

[a] During treatment, the abbreviated descriptors were recorded in the patient's chart, but they were codified using the associated score shown to better facilitate statistical analysis.
[b] Instances when two descriptors were recorded, the average score of the listed descriptors were used (i.e. CS/CG = 5.5 and CG/MIN = 4.5).
[c] In the mFIM, the original FIM category of Minimal Assistance (#4) has been sub-divided into Minimal Assistance (#4) and Contact Guard Assistance (#5).
[d] In the mFIM, the original FIM category of Supervision (#5) has been sub-divided into Close Supervision (#6), Supervision (#7), and Distant Supervision (#8).

Supplemental Table 2. Šídák's multiple comparisons test of perturbation level progression.

Session comparison	N1	N2	Mean 1 [a]	Mean 2 [a]	Mean Diff	t	DF	Adjusted P
1 vs 2	14	14	1.86	3.14	-1.29	4.08	90.00	0.0027
1 vs 3	14	14	1.86	4.43	-2.57	8.17	90.00	<0.0001
1 vs 4	14	14	1.86	4.93	-3.07	9.75	90.00	<0.0001
1 vs 5	14	14	1.86	5.57	-3.71	11.80	90.00	<0.0001
1 vs 6	14	13	1.86	6.14	-4.28	13.31	90.00	<0.0001
1 vs 7	14	14	1.86	6.50	-4.64	14.75	90.00	<0.0001
1 vs 8	14	14	1.86	6.86	-5.00	15.88	90.00	<0.0001
2 vs 3	14	14	3.14	4.43	-1.29	4.08	90.00	0.0027
2 vs 4	14	14	3.14	4.93	-1.79	5.67	90.00	<0.0001
2 vs 5	14	14	3.14	5.57	-2.43	7.71	90.00	<0.0001
2 vs 6	14	13	3.14	6.14	-3.00	9.32	90.00	<0.0001
2 vs 7	14	14	3.14	6.50	-3.36	10.66	90.00	<0.0001
2 vs 8	14	14	3.14	6.86	-3.71	11.80	90.00	<0.0001
3 vs 4	14	14	4.43	4.93	-0.50	1.59	90.00	0.9681
3 vs 5	14	14	4.43	5.57	-1.14	3.63	90.00	0.0131
3 vs 6	14	13	4.43	6.14	-1.71	5.32	90.00	<0.0001
3 vs 7	14	14	4.43	6.50	-2.07	6.58	90.00	<0.0001
3 vs 8	14	14	4.43	6.86	-2.43	7.71	90.00	<0.0001
4 vs 5	14	14	4.93	5.57	-0.64	2.04	90.00	0.7173
4 vs 6	14	13	4.93	6.14	-1.21	3.77	90.00	0.0083
4 vs 7	14	14	4.93	6.50	-1.57	4.99	90.00	<0.0001
4 vs 8	14	14	4.93	6.86	-1.93	6.13	90.00	<0.0001
5 vs 6	14	13	5.57	6.14	-0.57	1.77	90.00	0.9053
5 vs 7	14	14	5.57	6.50	-0.93	2.95	90.00	0.1077
5 vs 8	14	14	5.57	6.86	-1.29	4.08	90.00	0.0027
6 vs 7 [b]	13	14	6.14	6.50	-0.36	1.12	90.00	0.9998
6 vs 8 [b]	13	14	6.14	6.86	-0.72	2.23	90.00	0.5516
7 vs 8 [b]	14	14	6.50	6.86	-0.36	1.13	90.00	0.9998

DF, Degrees of Freedom

[a] Means are representative of the average perturbation level for the indicated session

[b] No significant differences were observed between session 6, 7, and 8

Supplemental Table 3. Šídák's multiple comparisons test of BBS scores: In-group comparisons.

Pre vs Post	N1	N2	Mean 1 (pre)	Mean 2 (post)	Mean Diff	t	DF	Adjusted P
SOC	30	30	40.20	50.50	-10.30	9.10	56.00	<0.0001
BWSS	15	15	30.20	45.27	-15.07	9.41	56.00	<0.0001
BWSS-P	14	14	30.43	48.29	-17.86	10.78	56.00	<0.0001

ANOVA; analysis of variance BWSS, body-weight support system; BWSS-P, body-weight support system with perturbations; DF, Degrees of Freedom; SD, Standard Deviation; SOC, Standard of Care.

Supplemental Table 4. Šídák's multiple comparisons test of BBS scores: Between-group comparisons.

Pre-Score	N1	N2	Mean 1	Mean 2	Mean Diff	t	DF	Adjusted P
SOC vs BWSS	30	15	40.20	30.20	10.00	4.64	112.0	<0.0001
SOC vs BWSS-P	30	14	40.20	30.43	9.77	4.43	112.0	<0.0001
BWSS vs BWSS-P	15	14	30.20	30.43	-0.23	0.09	112.0	0.9996
**Post-Score**	**N1**	**N2**	**Mean 1**	**Mean 2**	**Mean Diff**	**t**	**DF**	**Adjusted P**
SOC vs BWSS	30	15	50.50	45.27	5.23	2.43	112.0	0.0494
SOC vs BWSS-P	30	14	50.50	48.29	2.21	1.00	112.0	0.6821
BWSS vs BWSS-P	15	14	45.27	48.29	-3.02	1.19	112.0	0.5535

ANOVA; analysis of variance BWSS, body-weight support system; BWSS-P, body-weight support system with perturbations; DF, Degrees of Freedom; SD, Standard Deviation; SOC, Standard of Care.

Supplemental Table 5. Šídák's multiple comparisons test of Ambulation scores: In-group comparisons.

Pre vs Post	N1	N2	Mean 1 (pre)	Mean 2 (post)	Mean Diff	t	DF	Adjusted P
BWSS	15	15	4.33	7.80	-3.47	11.66	27.00	<0.0001
BWSS-P	14	14	4.75	8.64	-3.89	12.65	27.00	<0.0001

ANOVA; analysis of variance BWSS, body-weight support system; BWSS-P, body-weight support system with perturbations; DF, Degrees of Freedom; SD, Standard Deviation

Supplemental Table 6. Šídák's multiple comparisons test of Ambulation scores: Between-group comparisons.

Pre-Score	N1	N2	Mean 1	Mean 2	Mean Diff	t	DF	Adjusted P
BWSS vs BWSS-P	15	14	4.33	4.75	-0.42	1.12	54.00	0.4650
**Post-Score**	**N1**	**N2**	**Mean 1**	**Mean 2**	**Mean Diff**	**t**	**DF**	**Adjusted P**
BWSS vs BWSS-P	15	14	7.80	8.64	-0.84	2.26	54	0.0548

ANOVA; analysis of variance BWSS, body-weight support system; BWSS-P, body-weight support system with perturbations; DF, Degrees of Freedom; SD, Standard Deviation

Supplemental Table 7. Šídák's multiple comparisons test of Toilet Transfer scores: In-group comparisons.

Pre vs Post	N1	N2	Mean 1 (pre)	Mean 2 (post)	Mean Diff	t	DF	Adjusted P
BWSS	15	15	4.30	7.70	-3.40	11.39	27.00	<0.0001
BWSS-P	14	14	4.89	8.39	-3.50	11.33	27.00	<0.0001

ANOVA; analysis of variance BWSS, body-weight support system; BWSS-P, body-weight support system with perturbations; DF, Degrees of Freedom; SD, Standard Deviation

Supplemental Table 8. Šídák's multiple comparisons test of Toilet Transfer scores: Between-group comparisons.

Pre-Score	N1	N2	Mean 1	Mean 2	Mean Diff	t	DF	Adjusted P
BWSS vs BWSS-P	15	14	4.30	4.89	-0.59	1.73	54.00	0.1711
**Post-Score**	**N1**	**N2**	**Mean 1**	**Mean 2**	**Mean Diff**	**t**	**DF**	**Adjusted P**
BWSS vs BWSS-P	15	14	7.70	8.39	-0.69	2.02	54.00	0.0943

ANOVA; analysis of variance BWSS, body-weight support system; BWSS-P, body-weight support system with perturbations; DF, Degrees of Freedom; SD, Standard Deviation

Supplemental Table 9. Šídák's multiple comparisons test of ABC Scores: In-group comparisons.

Pre vs Post	N1	N2	Mean 1 (pre)	Mean 2 (post)	Mean Diff	t	DF	Adjusted P
BWSS	15	15	61.81	82.38	-20.56	4.10	26.00	0.0007
BWSS-P	14	14	63.88	84.81	-20.93	4.17	26.00	0.0006

ANOVA; analysis of variance BWSS, body-weight support system; BWSS-P, body-weight support system with perturbations; DF, Degrees of Freedom; SD, Standard Deviation

Supplemental Table 10. Šídák's multiple comparisons test of ABC scores: Between-group comparisons.

Pre-Score	N1	N2	Mean 1	Mean 2	Mean Diff	t	DF	Adjusted P
BWSS vs BWSS-P	15	14	61.81	63.88	-2.07	0.31	52.00	0.9409
**Post-Score**	**N1**	**N2**	**Mean 1**	**Mean 2**	**Mean Diff**	**t**	**DF**	**Adjusted P**
BWSS vs BWSS-P	15	14	82.38	84.81	-2.437	0.37	52.00	0.9189

ANOVA; analysis of variance BWSS, body-weight support system; BWSS-P, body-weight support system with perturbations; DF, Degrees of Freedom; SD, Standard Deviation

CONSORT 2010 checklist of information to include when reporting a randomised trial*

Section/Topic	Item No	Checklist item	Reported on page No
**Title and abstract**			
	1a	Identification as a randomised trial in the title	**n/a**
	1b	Structured summary of trial design, methods, results, and conclusions (for specific guidance see CONSORT for abstracts)	**p. 1-2**
**Introduction**			
Background and objectives	2a	Scientific background and explanation of rationale	**p. 2-3**
	2b	Specific objectives or hypotheses	**p. 4**
**Methods**			
Trial design	3a	Description of trial design (such as parallel, factorial) including allocation ratio	**p. 4**
	3b	Important changes to methods after trial commencement (such as eligibility criteria), with reasons	**p. 7-8 (line 190-195)**
Participants	4a	Eligibility criteria for participants	**p. 4-5, Table 1**
	4b	Settings and locations where the data were collected	**p. 4**
Interventions	5	The interventions for each group with sufficient details to allow replication, including how and when they were actually administered	**p. 8-10**
Outcomes	6a	Completely defined pre-specified primary and secondary outcome measures, including how and when they were assessed	**p. 6-8**
	6b	Any changes to trial outcomes after the trial commenced, with reasons	**n/a**
Sample size	7a	How sample size was determined	**p. 6, Figure 1**
	7b	When applicable, explanation of any interim analyses and stopping guidelines	**n/a**
Randomisation:			
Sequence generation	8a	Method used to generate the random allocation sequence	**p. 4**
	8b	Type of randomisation; details of any restriction (such as blocking and block size)	**p. 4, 6 (line 152), Figure 1**
Allocation concealment mechanism	9	Mechanism used to implement the random allocation sequence (such as sequentially numbered containers), describing any steps taken to conceal the sequence until interventions were assigned	**p. 4**
Implementation	10	Who generated the random allocation sequence, who enrolled participants, and who assigned participants to interventions	**p. 6 (line 152-153)**
Blinding	11a	If done, who was blinded after assignment to interventions (for example, participants, care providers, those assessing outcomes) and how	**n/a**

	11b	If relevant, description of the similarity of interventions	**p. 8-10**
Statistical methods	12a	Statistical methods used to compare groups for primary and secondary outcomes	**p. 10-12**
	12b	Methods for additional analyses, such as subgroup analyses and adjusted analyses	**p. 10-12**
**Results**			
Participant flow (a diagram is strongly recommended)	13a	For each group, the numbers of participants who were randomly assigned, received intended treatment, and were analysed for the primary outcome	**p. 12-13 Figure 1**
	13b	For each group, losses and exclusions after randomisation, together with reasons	**p. 12-13 Figure 1**
Recruitment	14a	Dates defining the periods of recruitment and follow-up	**p. 5**
	14b	Why the trial ended or was stopped	**n/a**
Baseline data	15	A table showing baseline demographic and clinical characteristics for each group	**p. 13, Table 2**
Numbers analysed	16	For each group, number of participants (denominator) included in each analysis and whether the analysis was by original assigned groups	**p. 12-13, Figure 1, Figure Legends**
Outcomes and estimation	17a	For each primary and secondary outcome, results for each group, and the estimated effect size and its precision (such as 95% confidence interval)	**p. 13-17, Table 3, Figure 2-4**
	17b	For binary outcomes, presentation of both absolute and relative effect sizes is recommended	**p. 13-17, Table 3, Figure 2-4**
Ancillary analyses	18	Results of any other analyses performed, including subgroup analyses and adjusted analyses, distinguishing pre-specified from exploratory	**p. 13-17, Table 2-3, Figure 2-4, Sup. Materials**
Harms	19	All important harms or unintended effects in each group (for specific guidance see CONSORT for harms)	**p. 12-13**
**Discussion**			
Limitations	20	Trial limitations, addressing sources of potential bias, imprecision, and, if relevant, multiplicity of analyses	**p. 17-22**
Generalisability	21	Generalisability (external validity, applicability) of the trial findings	**p. 17-22**
Interpretation	22	Interpretation consistent with results, balancing benefits and harms, and considering other relevant evidence	**p. 17-22**
**Other information**			
Registration	23	Registration number and name of trial registry	**p. 2, 5**
Protocol	24	Where the full trial protocol can be accessed, if available	**p. 23**
Funding	25	Sources of funding and other support (such as supply of drugs), role of funders	**p. 23**
